# Technical and Diagnostic Issues in Whole Slide Imaging Published Validation Studies

**DOI:** 10.3389/fonc.2022.918580

**Published:** 2022-06-16

**Authors:** Paola Chiara Rizzo, Ilaria Girolami, Stefano Marletta, Liron Pantanowitz, Pietro Antonini, Matteo Brunelli, Nicola Santonicco, Paola Vacca, Nicola Tumino, Lorenzo Moretta, Anil Parwani, Swati Satturwar, Albino Eccher, Enrico Munari

**Affiliations:** ^1^ Department of Pathology and Diagnostics and Public Health, Section of Pathology, University Hospital of Verona, Verona, Italy; ^2^ Division of Pathology, Central Hospital, Bolzano, Italy; ^3^ Department of Pathology, Pederzoli Hospital, Peschiera del Garda, Italy; ^4^ Department of Pathology & Clinical Labs, University of Michigan, Ann Arbor, MI, United States; ^5^ Bambino Gesù Children’s Hospital, Istituto di Ricovero e Cura a Carattere Scientifico (IRCCS), Rome, Italy; ^6^ Department of Pathology, Ohio State University Medical Center, Columbus, OH, United States; ^7^ Department of Pathology and Diagnostics, University and Hospital Trust of Verona, Verona, Italy; ^8^ Department of Molecular and Translational Medicine, University of Brescia, Brescia, Italy

**Keywords:** whole slide imaging, digital pathology, validation study, systematic (literature) reviews, artificial intelligence

## Abstract

**Objective:**

Digital pathology with whole-slide imaging (WSI) has many potential clinical and non-clinical applications. In the past two decades, despite significant advances in WSI technology adoption remains slow for primary diagnosis. The aim of this study was to identify common pitfalls of WSI reported in validation studies and offer measures to overcome these challenges.

**Methods:**

A systematic search was conducted in the electronic databases Pubmed-MEDLINE and Embase. Inclusion criteria were all validation studies designed to evaluate the feasibility of WSI for diagnostic clinical use in pathology. Technical and diagnostic problems encountered with WSI in these studies were recorded.

**Results:**

A total of 45 studies were identified in which technical issues were reported in 15 (33%), diagnostic issues in 8 (18%), and 22 (49%) reported both. Key technical problems encompassed slide scan failure, prolonged time for pathologists to review cases, and a need for higher image resolution. Diagnostic challenges encountered were concerned with grading dysplasia, reliable assessment of mitoses, identification of microorganisms, and clearly defining the invasive front of tumors.

**Conclusion:**

Despite technical advances with WSI technology, some critical concerns remain that need to be addressed to ensure trustworthy clinical diagnostic use. More focus on the quality of the pre-scanning phase and training of pathologists could help reduce the negative impact of WSI technical difficulties. WSI also seems to exacerbate specific diagnostic tasks that are already challenging among pathologists even when examining glass slides with conventional light microscopy.

## Introduction

Virtual microscopy (VM) using digital whole slide imaging (WSI) is a technology by which glass slides in pathology are digitally scanned at high-resolution for viewing on a computer screen. Ever since WSI scanners first became commercially available around two decades ago, progress in the technology of these devices has continually improved their image resolution, image quality, slide throughput, end-user software tools, and integration with laboratory information systems (1). Applications of WSI for clinical (e.g. telepathology, quantitative image analysis) and non-clinical (e.g. education and research) have markedly increased (2–6). Ample literature has been published demonstrating excellent concordance between utilizing WSI versus glass slides with traditional light microscopy (LM) to render diagnoses (7, 8). Nevertheless prior to implementing WSI for diagnostic use in clinical practice, several associations have recommended that such technology be validated by pathology laboratories for their intended use (9). Recently, the College of American Pathologists (CAP) updated their guideline providing recommendations for validating WSI for primary diagnosis (10). The validation process should “stress test” the WSI system in the appropriate clinical environment in order to assess that it allows pathologists to accurately diagnose cases, at least at the same level of accuracy as LM, and to identify and control for potential interfering artifacts or technological risks that could impair patient safety (10, 11).

Whilst published validation studies have largely focused on the success of WSI for specific clinical use cases, some of the “negative issues” that were encountered including technical failures or particular diagnostic difficulties were often under-reported. Furthermore, only few systematic analyses on this topic devoted to the tribulations of employing WSI in clinical practice have been performed. In the literature review undertaken by Goacher et al. from 2017, for example, the authors reported that there was in fact a slower time to diagnosis when using WSI compared with LM (7). The aim of this study was to systematically review the literature of published validation studies that evaluated the feasibility of WSI for diagnostic clinical use in pathology, recording and subsequently analyzing any technical and/or diagnostic problems encountered.

## Material and Methods

A systematic review of the literature was conducted according to the guideline for Preferred Reporting Items for a Systematic Review and Meta-Analysis (12).

Electronic searches were carried out in the databases PubMed-MEDLINE and Embase until the 5th of December, 2021. No study type filters were used nor language restriction applied. References listed in all identified studies were also hand-searched to retrieve potential additional studies. Initial screening of articles by title/abstract was performed with the aid of the online systematic review web-app QRCI (13). Eligibility of published studies was determined independently by two reviewers with disagreement resolved through consensus. Inclusion criteria included the details of a validation study with a series of surgical pathology cases assessed with WSI and with LM, not only reporting concordance data but also noting any negative issues encountered during the validation process. Studies represented only by abstracts were excluded, as were reviews and published letters to the editor with no original data. Data extracted included: authors, year published, country of study, number and type of cases selected, critical issues reported divided ac-cording to issues pertaining to diagnostic and technical problems. Specific technical problems searched for included slide scan failures, delayed scan time, and difficulties related to viewing and navigating digital slides.

## Results

**Figure 1:**
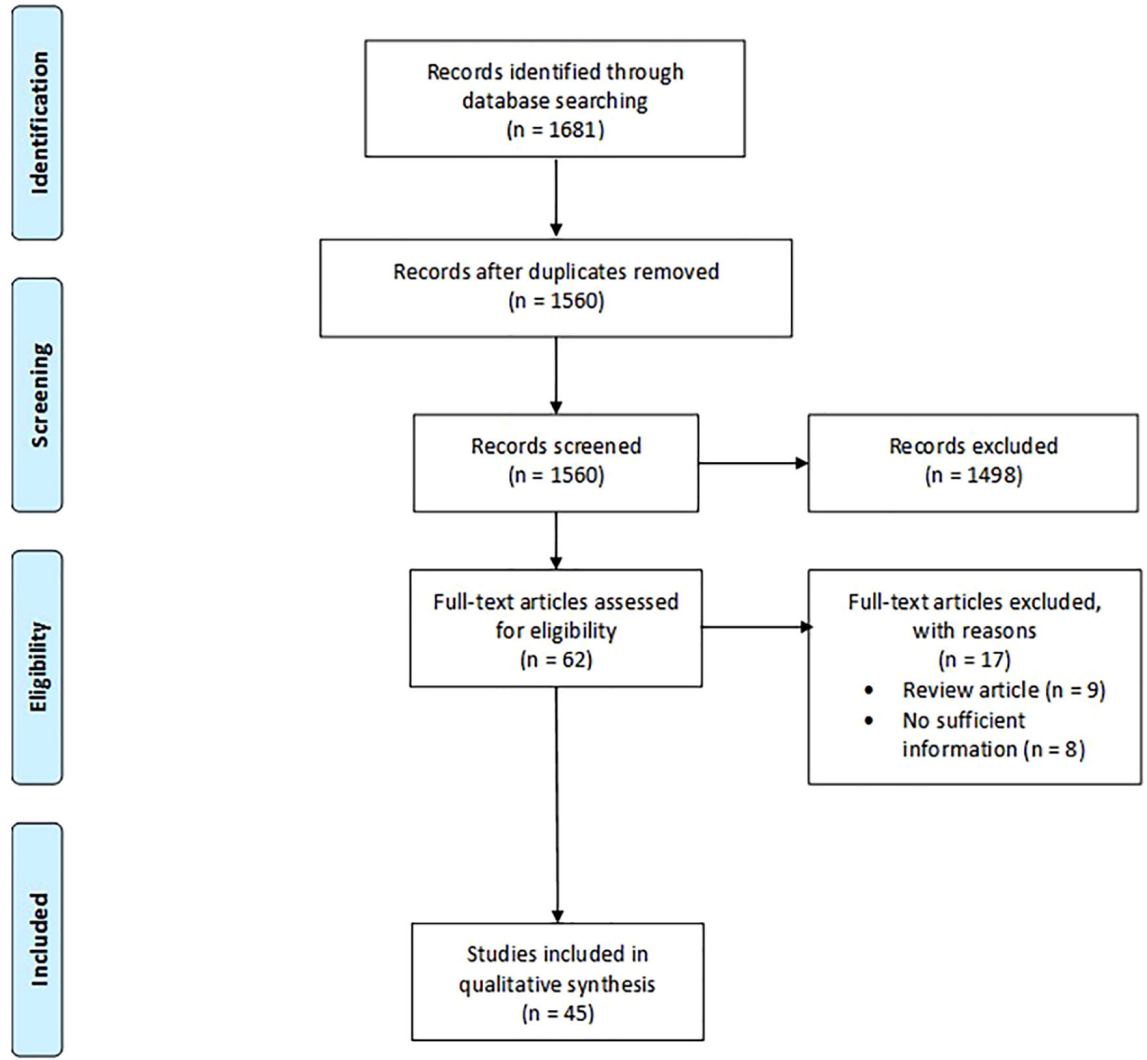
Search flow diagram. The diagram was designed according to the template of the PRISMA flow diagram from Page et al. (12) available at the PRISMA website (www.prismastatement.org).

The search strategy identified a total of 1560 records, with only 45 suitable articles finally included in our analysis (**Figure 1**). Publication dates ranged from 2007 to 2021. Twenty (45%) of the included studies were from North American countries, nineteen (42%) were from European countries and six (13%) were from non-European and non-North American countries. In more than half of the included studies (n=24, 57%) the participating pathologists were experienced in digital pathology. The length of the washout period between LM and WSI diagnosis ranged from 7 days to 2 years, but 13 studies did not report any washout time. The number of cases in the included studies varied from 23 to 3222. Twenty-two studies included cases from various pathology subspecialties, while 23 selected a specific diagnostic field. The majority of Authors used Aperio scanners (n=18, 40%), followed by Ventana scanners (n=6, 13%), NanoZoomer (n=5, 11%), MIRAMAX (n=3, 7%), Philips (n=3, 7%), Pannoramic (n=2, 4%), DHistech (n=2, 4%), NAVIGO (n=1, 3%), Grandium Ocus (n=1, 1%), and OMYX (n=1, 3%). Twelve of the viewer systems used in these studies were APERIO (26%), 5 Ventana (11%), 3 Leica (7%), 3 DHistech (7%), 3 Philips (7%), 2 PathXL (4%), 2 Pannoramic (4%), 1 OMYX (3%), 1 Grandium Ocus (3%), and 1 CaloPix (3%). Only one study tested the use of tablets, specifically the iPad (14). Only one study tested the use of tablets, specifically the iPad (14).

In order to summarize the pitfalls documented in the various validation studies, we categorized all the discordances into two main groups: technical issues and diagnostic issues (**Table 1**).

**Table 1:** Characteristics of technical and diagnostic issues of the included studies.

Technical issue	n (%)	Diagnostic issue	n (%)
Timing (scanning, viewing)	19 (42%)	Grade of dysplasia	8 (18%)
Scanning failure	9 (20%)	Mitotic count	6 (13%)
Need for higher magnification	9 (20%)	Misinterpretation of diagnosis	4 (9%)
Storage	7 (15%)	Lack of confidence	3 (6%)
Lack of multiple focus planes	8 (18%)	Identification of microorganisms	3 (6%)
Color inaccuracy	3 (6%)	Legal issues	2 (4%)
Difficulty using mouse	2 (4%)	Misinterpretation of inflammatory cells	2 (4%)
Need for polarization	2 (4%)	Identification of tumor invasion	2 (4%)
Underexposure of images	1 (2%)	Misinterpretation of fibrosis	1 (2%)
Server	1 (2%)	Misinterpretation of intraepithelial lymphocytes	1 (2%)
Workstation ergonomics	1 (2%)	Misinterpretation of calcification and focal atypia	1 (2%)
Presence of artifacts	1 (2%)	Overestimation of blasts’ count	1 (2%)

### Technical Issues

Sixteen (36%) studies reported about technical issues only, eight (18%) reported on diagnostic issues only, and 21 (46%) reported both on technical and diagnostic issue. Among the technical issues described, nine studies (20%) reported failures in scanning glass slides, 19 studies (42%) considered WSI more time consuming than LM, and nine studies (20%) reported specifically the need for higher magnification (better image resolution) with WSI to more easily view and navigate cases. Other technical issues reported were: lack of focus (n=8, 18%), suboptimal navigation tools (n=2, 4%), need for polarization (n=2, 4%), and lack of adequate color fidelity for special stain or immunohistochemical staining (n=3, 7%). In addition, some validation studies (n=7, 15%) reported difficulty related to image storage.

### Diagnostic Issues

Concerning diagnostic issues when using WSI, in eight studies (18%) grading of dysplasia represented the most common problem encountered. Furthermore, six (13%) studies re-ported challenges in assessing mitotic count, four (9%) studies reported general misdiagnosis, while three (7%) studies reported discordant diagnoses related to the identification of microorganisms. In three (7%) studies the authors mentioned there was lack of diagnostic confidence, and in two (4%) that pathologists experienced difficulty interpreting the invasive component of tumors.

The characteristics of each of the included studies are extensively detailed in **Supplementary Table S1**.

## Discussion

Digital pathology has been increasingly deployed in many institutions (15, 16). Nevertheless, problems encountered when using WSI for routine pathology diagnosis still remain. A critical appraisal of such issues is important to understand and hopefully re-solve. Our systematic review identified 45 articles that specifically reported problems experienced with WSI usage for primary diagnosis during a validation process.

As expected, technical issues when validating WSI were the most frequently reported. The most commonly mentioned technical issue involved scan failures with the need for re-scan slides and the consequent prolonging of turn-around-time (TAT). When combined with the reported experience by participating pathologists that it took them longer to evaluate digital slides in order to establish a diagnosis, switching to WSI for primary diagnosis has the potential to delay TAT. This drawback would need to be offset by some of the other workflow benefits of digital pathology such as decreased time for slide distribution, quicker archival image retrieval, in addition to ensuring faster network connections, better workstations and improved viewing software.

Newer scanners with higher throughput capacity and reduced image acquisition time have further helped overcome TAT issues. The quality of pre-imaging factors can also help re-duce the aforementioned limitation of delayed TAT. For example, striving to produce uniform histological sections without folds and clean, dry slides without artifacts such as air bubbles are important to reduce the probability of scan failures. Such pre-imaging measures are especially important for the digitization of cytology slides (17, 18), where thick smears, three dimensional cell groups and obscuring material make it harder for scanners without z-stacking capability to achieve optimal focus. For some studies, the technical difficulty noted when viewing digital slides was related to the monitor and input device (specifically, computer mouse) used. Hanna et al. (19) suggested trying different input devices instead of a conventional mouse to circumvent problems with digital slide navigation. Similarly, Brunelli et al. (14) tested the use of a tablet to improve WSI navigation. Although a validation study should not suffice for official training of end-users, spending more time training pathologists to better use WSI and allowing them to become more familiar with this technology can certainly improve their ease with utilizing WSI. Alassiri et al. (20) showed that at the beginning of their validation study participating pathologists were slow-er in assessing cases with WSI, but by the end of their validation process they experienced no notable time difference when reading cases with either WSI or LM modalities.

The other important category of shortcomings with WSI that emerged in published validation studies was concerned with performing certain challenging diagnostic tasks. The most frequently reported were misinterpretation regarding grading of dysplasia, in a variety of settings including gastrointestinal biopsies (21–23) and melanocytes atypia (24). Such errors were related to both downgrading or upgrading lesions (25–30). Apart from the cited discordance related to interpreting gastrointestinal dysplasia, other challenging diagnostic areas that were reported in validation studies included urothelial dysplasia, cervical dysplasia, grading of ovarian and endometrial cancers, *in situ* lesions of the breast, and brain pathology. However, in most studies, especially the most recent publications, overall diagnostic concordance was above the cutoff of 95% recommended in the CAP guidelines for WSI vali-dation, and discordances with a potential impact on clinical management were often lower than 3% (8, 10, 21, 29–31). Another frequently reported area of discordance, as well source of dissatisfaction among pathologists, when using WSI relates to counting mitoses, such as is required in grading meningiomas (32) or breast carcinoma (33, 34) or when diagnosing malignancy in a melanocytic lesion (35). Other less frequently reported. but still relevant, reported diagnostic difficulties with WSI were the detection of microorganisms (19), discriminating single inflammatory cell types in dermatopathology and hematopathology (24, 36–38), assessment of tumor budding and tumor pattern of invasion in colon cancer (39), and overestimation of steatosis and fibrosis in liver cases (40). In general, pathologists reported lower diagnostic confidence when signing out with WSI. Similar considerations have also been observed in the setting of pediatric pathology (41), where WSI showed to be at least as reliable as LM, fully satisfying the CAP guidelines. Even in this setting the few reported discrepancies concerned subtle morphological features, such as identification of Candida spores and hypha, likely linked to the difficult of the case rather than to the classic or digital method of evaluation of the slides. Many of these diagnostic concerns are being addressed with improvements in WSI technology (e.g. incorporating higher resolution cameras and objectives into scanners) and leveraging artificial intelligence (AI) to apply algorithms for specific (narrow) tasks such as counting mitoses, screening slides for microorganisms, and standardizing the grading of dysplasia or cancer. Development and deployment of these technologies are foreseeably going to increase in the near future, further allowing pathologists to benefit from digital supports to proficiently reach proper histological diagnoses for both adults and pediatric patients.

Additional collateral problems were also reported in published validation studies, which were mainly of a technical and institution’s organizational nature. Some authors reported problems related to the storage of WSI cases, given the huge size of WSI files and consequent high demand this has on information technology (IT) infra-structure for image management (31, 37, 42, 43). Lastly, Ordi et al. (44) and Al-Janabi et al. (43) reported about the cultural barrier of pathologists, including their concerns about legal is-sues and resistant mindset to accept WSI over more familiar LM for routine primary diagnosis. However, since then we have witnessed increased regulatory approval of WSI solutions, such as clearances issued by the Food and Drug Administration (FDA) in the USA (45, 46) for primary diagnosis which has helped increase overall confidence in the adoption of WSI. Moreover, with the rapid shift experienced towards using digital pathology to permit re-mote signing-out during the Coronavirus Disease 2019 (COVID-19) pandemic many pre-pandemic skeptics have since been convinced about the value of digital pathology (47).

## Conclusions

Digital pathology with WSI is nowadays a reality in many laboratories, but there are still some negative aspects that may restrain an even wider spread adoption of WSI. When reviewing the literature for validation studies highlighting these conflicting aspects, we found both some technical and diagnostic critical issues still remain of concern. The majority of technical points could be reasonably overcome by further improvement of technology and dedicated training of pathologists. Likewise, the diagnostic issues are mainly represented by subtle tasks which yield per se an unsatisfactory reproducibility among pathologists with conventional glass slides as well. In the near future, the development of dedicated and more objective AI tools could be of aid to further support pathologists in reducing the gap between LM and WSI in order to increase the efficiency of the diagnostic process and ultimately improve patients’ management and care.

## Data Availability Statement

The original contributions presented in the study are included in the article/**Supplementary Material**. Further inquiries can be directed to the corresponding author.

## Author Contributions

Conceptualization, IG, SM, LP and AE. Data curation, PR, IG, SM and AE. Formal analysis, PR, IG and SM. Methodology, IG, LP and AE. Project administration, AE and EM. Supervision, AE and EM. Validation, PA, MB, NS, PV, NT, LM, and EM. Writing – original draft, PRC, IG and SM. Writing – review & editing, PA, MB, NS, PV, NT, LM, AP, SS, AE and EM. All authors contributed to the article and approved the submitted version.

## Funding

This work was supported by grants from Associazione Italiana Ricerca sul Cancro (AIRC) Investigator Grant ID 19920 (LM); Special Program Metastatic disease: the key unmet need in oncology 5 per mille 2018, ID 21147 (LM).

## Conflict of Interest

The authors declare that the research was conducted in the absence of any commercial or financial relationships that could be construed as a potential conflict of interest.

## Publisher’s Note

All claims expressed in this article are solely those of the authors and do not necessarily represent those of their affiliated organizations, or those of the publisher, the editors and the reviewers. Any product that may be evaluated in this article, or claim that may be made by its manufacturer, is not guaranteed or endorsed by the publisher.
