# Supplementary material for: Technical and Diagnostic Issues in Whole Slide Imaging Published Validation Studies
Source: Front Oncol. 2022 Jun 16;12:918580. doi: 10.3389/fonc.2022.918580 (PMC9246412; doi:10.3389/fonc.2022.918580)
Supplement: Supplementary file 1 [file Table_1.docx]

Supplementary table 1: detailed technical and diagnostic issues reported in the included studies.

| **Author, year, Country** | **Pathology** | **N cases** | **Scanner** | **Viewing software** | **Technical issues** | | | | | | **Diagnostic issues** | | | | | |
| --- | --- | --- | --- | --- | --- | --- | --- | --- | --- | --- | --- | --- | --- | --- | --- | --- |
|  |  |  |  |  | **scanning fail** | **Time issues (scanning viewing)** | **Need for higher magnification** | **Storage** | **Lack of multiple focus planes** | **Other technical issues** | **Grade of dysplasia** | **Lack of confidence** | **Mitotic count** | **Misinterpretation of diagnosis** | **Identification of microorganisms** | **Other diagnostic issues** |
| Fine, 2007,  USA | genitourinary | 30 | T2, Aperio Technologies, Vista, CA | na |  | ✓ |  |  |  |  |  |  |  |  |  |  |
| Velez, 2008,  USA | dermatology | 45 | T2, Aperio Technologies, Vista, CA | na | ✓ |  |  |  | ✓ |  | ✓ |  | ✓ |  |  |  |
| Nielsen, 2010,  Denmark | dermatology | 96 | Mirax Scan | MIRAX viewer |  |  |  |  | ✓ |  |  |  |  | ✓ |  |  |
| Al-Janabi, 2011,  The Netherlands | dermatology | 100 | ScanScope XT scanners (Aperio, Vista, California, USA) | Aperio ScanScope XT |  | ✓ | ✓ | ✓ |  |  |  |  |  |  |  | legal issue |
| Al-Janabi, 2011,  The Netherlands | gastrointestinal | 100 | ScanScope XT scanners (Aperio, Vista, California, USA) | Aperio ScanScope XT |  |  | ✓ | ✓ |  | Difficulty using mouse |  |  |  |  |  |  |
| Gui, 2012,  USA | gastrointestinal | 42 | Aperio XT scanner (Aperio Technologies, Vista, CA) | Aperio ScanScope XT |  | ✓ |  |  |  |  | ✓ |  |  |  |  |  |
| Campbell, 2012,  USA | miscellaneous | 312 | 3 iScan instruments (BioImagene Medical Systems, Inc, Sunnyvale, CA) | Virtuoso Web-based viewing software | ✓ |  |  |  |  |  |  |  |  |  | ✓ |  |
| Al-Janabi, 2012, The Netherlands | breast | 100 | ScanScope XT scanners (Aperio, Vista, CA) | Aperio ScanScope XT |  |  | ✓ |  |  |  |  |  |  |  |  |  |
| Al-Janabi, 2012, The Netherlands | miscellaneous | 3222 | Mirax Scan | na | ✓ | ✓ |  | ✓ | ✓ |  |  |  |  |  |  |  |
| Fònyad, 2012, Hungary | miscellaneous | 306 | 3Dhistech (3DH) Scan 1.11 | 3DH DataBase |  |  |  |  | ✓ | Color inaccuracy |  |  |  |  |  | interpreting lymph nodes |
| Bauer, 2013, USA | miscellaneous | 607 | Leica Biosystems, Aperio CS scanner | ePathAccess, Leica Biosystems |  |  | ✓ |  |  | Color inaccuracy |  |  |  |  |  | misinterpretation of inflammatory cells |
| Krishnamurthy, 2013, USA | breast | 150 | Aperio Digital Pathology System | Aperio Digital Pathology System |  | ✓ |  |  |  |  |  |  |  |  |  |  |
| Al-Janabi, 2014, The Netherlands | genitourinary | 100 | na | na |  | ✓ | ✓ |  |  |  |  |  |  |  |  |  |
| Campbell, 2014,  USA | breast | 85 | Ventana iCoreo scanner | Ventana Virtuoso Express viewing software (Ventana Medical Systems, Tucson, AZ) |  |  |  |  |  |  | ✓ |  |  |  |  |  |
| Bauer, 2014, USA | miscellaneous | 217 | ScanScope XT, Aperio, Vista, California | ImageScope, Aperio |  |  |  |  |  |  | ✓ |  |  |  |  |  |
| Brunelli, 2014, Italy | miscellaneous | 61 | Scan- Scope Digital Slides Scanner | ImageScope, Aperio |  |  | ✓ |  |  |  |  |  |  |  |  | legal issue |
| Houghton, 2014 UK | miscellaneous | 100 | Hamamatsu Nanozoomer (Hamamatsu UK). | pathXL cloud platform (PathXL, UK) |  | ✓ |  |  |  |  |  |  |  |  |  |  |
| Thrall, 2014,  USA | miscellaneous | 100 | iScan Coreo Au | Virtuoso viewing software |  | ✓ |  |  | ✓ |  |  |  |  |  |  |  |
| Gage, 2014,  USA | genitourinary | 500 | ScanScope XT | Aperio ImageScope reader |  |  |  | ✓ |  |  |  |  |  |  |  |  |
| Ordi, 2014,  Spain | gynaecology | 452 | Ventana iScan HT (Roche diagnostics) | Virtuoso viewer (Roche) |  |  |  | ✓ |  |  |  | ✓ |  |  |  |  |
| Bauer, 2015,  USA | miscellaneous | 75 | na | eSlideManager Healthcare Network, Leica Biosystems |  | ✓ |  |  |  |  | ✓ |  |  |  |  |  |
| Thrall,2015,  USA | miscellaneous | 1000 | iScan Coreo Au whole slide scanners | Virtuoso viewing software |  | ✓ | ✓ |  |  | server |  |  |  | ✓ | ✓ |  |
| Loughrey, 2015,  UK | gatrointestinal | 100 | Hamama- tsu Nanozoomer (Hamamatsu, United Kingdom) | PathXL (PathXL, United Kingdom) |  |  |  |  |  | underexpose image | ✓ |  |  |  |  | epithelial lymphocytes |
| Snead, 2016,  UK | miscellaneous | 3017 | Omnyx VL4 scanner (LLC 1251; Omnyx, Pittsburgh, PA, USA) | Omnyx Integrated Digital pathology (IDP) | ✓ |  | ✓ |  |  | Color inaccuracy, need for polarization |  |  |  |  | ✓ |  |
| Fertig, 2017,  USA | dermatology | 40 | MIRAX MIDI slide scanner (Carl Zeiss AG, Oberkochen, Germany) | Pannoramic Viewer Software (3DHISTECH) |  | ✓ |  |  |  |  |  |  |  |  |  | misinterpretation of inflammatory cells |
| Tabata, 2017,  Japan | miscellaneous | 900 | 6 different scanners | na |  | ✓ |  | ✓ |  |  |  |  |  |  |  |  |
| Villa, 2018,  France | miscellaneous | 119 | NanoZoomer HT C9600 | CaloPix |  |  |  |  |  | workstation ergonomics | ✓ |  |  | ✓ |  |  |
| Cima, 2018  Italy | miscellaneous | 121 | Navigo digital system (Visia Imaging) | Navigo” digital system (Visia Imaging) |  | ✓ |  |  |  |  |  |  | ✓ |  |  | Overestimation of steatosis and underestimation of fibrosis. |
| Rakha, 2018,  UK | breast | 1675 | 3D Histech Panoramic 250 Flash II scanner | 3D Histech Pannoramic Viewer |  |  |  |  |  |  |  |  | ✓ |  |  |  |
| Mukhopadhyay, 2018, USA | miscellaneous | 1992 | Philips IntelliSite Pathology Solution (Philips, the Netherlands) | Philips IntelliSite Pathology Solution |  |  |  |  |  |  |  |  |  | ✓ |  |  |
| Williams, 2018,  UK | breast | 694 | Aperio AT2 scanner | e-Slide Manager software Aperio | ✓ |  |  |  | ✓ | presence of artifacts |  |  | ✓ |  |  | identification of invasion tumor, calcification and focal atypia. |
| Davidson, 2019,  USA | breast | 22 | iScan Coreo Au | na |  |  |  |  |  |  |  |  | ✓ |  |  |  |
| Hanna, 2019,  USA | miscellaneous | 204 | Leica Aperio AT2 | Leica eSlide manager | ✓ | ✓ |  |  |  | Difficulty using mouse |  | ✓ |  |  |  |  |
| Sturm, 2019,  The Netherland | dermatology | 102 | Pannoramic 250 Flash II scanner | Pannoramic Viewer |  |  |  |  | ✓ |  |  |  | ✓ |  |  |  |
| Hanna, 2020, USA | miscellaneous | 108 | Aperio GT450 | Aperio GT450 |  | ✓ |  |  |  | Need of polarization |  |  |  |  |  |  |
| Borowsky, 2020, USA | miscellaneous | 2045 | Aperio AT2 DX system | ImageScope Aperio |  |  |  |  |  |  |  | ✓ |  |  |  |  |
| Rajaganesan, 2020, India | miscellaneous | 240 | na | na |  |  |  |  | ✓ | presence of artifacts |  |  |  |  |  |  |
| Barbosa Diniz, 2020, Brazil | ear-nose-throat | 25 | Aperio Scan- Scope CS (Aperio Technologies, Vista, CA, USA) | na |  | ✓ | ✓ |  |  |  |  |  |  |  |  |  |
| Hacking, 2020, USA | gastrointestinal | 231 | Leica Aperio AT2 (Leica Biosystems, Buffalo Grove, Illinois, USA) | Aperio viewer |  |  |  |  |  |  |  |  |  |  |  | identification of invasion tumor |
| Alassiri, 2020, Saudi Arabia | neurology | 60 | Aperio scanner (ScanScope AT Turbo) | na |  | ✓ |  |  |  |  |  |  |  |  |  |  |
| Van Den Brand, 2020,  The Netherland | hematology | 93 | Philips IntelliSite Ultra Fast Scanner  (Philips Digital Pathology, Best, the Netherlands) | Philips Image Management System |  | ✓ |  | ✓ | ✓ |  |  |  |  |  |  | overestimation of blasts |
| Ammendola, 2021, Italy | neurology | 35 | NanoZoomer S360 Digital slide Hamamatsu PhotonicsTM | na |  |  |  |  |  |  |  |  | ✓ |  |  |  |
| Kaushal, 2021 India | miscellaneous | 60 | Grundium Ocus, Finland | Grundium Ocus, Finland | ✓ | ✓ |  |  |  |  |  |  |  |  |  |  |
| Ramaswamy, 2021, India | miscellaneous | 80 | Philips UFS 300 (Ultrafast scanner 300) | Image Management System | ✓ |  |  |  |  |  |  |  |  |  |  |  |
| Samuelson, 2021, USA | miscellaneous | 171 | P1000 Pannoramic scanner (3DHistech) | CaseViewer 2.3.0 | ✓ |  |  |  |  |  | ✓ |  |  |  |  |  |

✓: issue present, na: not available
